# How can event attribution science underpin financial decisions on Loss and Damage?

**DOI:** 10.1093/pnasnexus/pgae277

**Published:** 2024-08-27

**Authors:** Dim Coumou, Paola A Arias, Ana Bastos, Charlotte Kendra Gotangco Gonzales, Gabriele C Hegerl, Pandora Hope, Christopher Jack, Friederike Otto, Fahad Saeed, Olivia Serdeczny, Theodore G Shepherd, Robert Vautard

**Affiliations:** Institute for Environmental Studies, Vrije Universiteit Amsterdam, De Boelelaan 1105, 1081 HV, Amsterdam, the Netherlands; Royal Netherlands Meteorological Institute (KNMI), Department of Weather and Climate Models, Utrechtseweg 297, 3731 GA, De Bilt, Netherlands; Institut Pierre-Simon Laplace (IPSL), French National Centre for Scientific Research, Université Paris-Saclay, Sorbonne Université, place Jussieu, 75252 Paris, France; Grupo de Ingeniería y Gestión Ambiental, Escuela Ambiental, Facultad de Ingeniería, Universidad de Antioquia, Calle 67, No 53-108, Medellín, Colombia; Max Planck Institute for Biogeochemistry, Department of Biogeochemical Integration, 07745 Jena, Germany; Leipzig University, Institute for Earth System Science and Remote Sensing, 04017 Leipzig, Germany; Ateneo Institute of Sustainability, Ateneo de Manila University, Loyola Heights, Quezon City 1108, Philippines; Department of Environmental Science, School of Science and Engineering, Ateneo de Manila University, Loyola Heights, Quezon City 1108, Philippines; School of GeoSciences, Edinburgh University, Drummond St, EH8 9XP, Edinburgh, United Kingdom; Bureau of Meteorology, Australian Government, 700 Collins St, Melbourne 3008, Australia; Climate System Analysis Group, University of Cape Town, Rondebosch 7701, Cape Town, South Africa; Grantham Institute, Faculty of Natural Sciences, Imperial College London, 32 Lincoln’s Inn Fields, WC2A 3PH, London, United Kingdom; Climate System Analysis Group, University of Cape Town, Rondebosch 7701, Cape Town, South Africa; Weather and Climate Services, 108,Lane 1, Lake View Lanes (LVL), Korang Road, Bani Gala, Islamabad, Pakistan; Climate System Analysis Group, University of Cape Town, Rondebosch 7701, Cape Town, South Africa; Geography Department, Humboldt Universität zu Berlin, Rudower Ch 16, 12489 Berlin, Germany; Department of Meteorology, University of Reading, Whiteknights Road, Earley Gate, RG6 6ET, Reading, United Kingdom; Institut Pierre-Simon Laplace (IPSL), French National Centre for Scientific Research, Université Paris-Saclay, Sorbonne Université, place Jussieu, 75252 Paris, France

**Keywords:** extreme weather, climate impact, loss and damage, attribution

## Abstract

With climate extremes hitting nations across the globe, disproportionately burdening vulnerable developing countries, the prompt operation of the Loss and Damage fund is of paramount importance. As decisions on resource disbursement at the international level, and investment strategies at the national level, loom, the climate science community's role in providing fair and effective evidence is crucial. Attribution science can provide useful information for decision makers, but both ethical implications and deep uncertainty cannot be ignored. Considering these aspects, we articulate a vision that integrates established attribution methods and multiple lines of evidence within a coherent logical framework.

## Introduction

In its latest assessment report (AR6), the Intergovernmental Panel on Climate Change (IPCC) concluded with high confidence that “human-caused climate change is already affecting weather and climate extremes in every region” and that this is leading to “widespread adverse impacts and related losses and damages” (1). The basis for this assessment builds primarily on advances in extreme weather attribution, linking (a class of) extreme events and their observed impacts to anthropogenic climate change. Indeed, the last few years have seen many examples including devastating floods in Pakistan (2), a record-shattering heat wave in Canada (3), and prolonged droughts in Europe (4) and South America (5). These extremes caused devastating losses and damages, and all had a clear climate change signal (6). For other events, such as the extreme rainfall and flooding in central Africa, data scarcity prevented drawing any conclusions about the role of climate change in these floods (7).

In the context of such events unfolding, COP27 (Conference of the Parties of the United Nations Framework Convention on Climate Change) established a fund to support vulnerable developing countries in dealing with Loss and Damage (L&D). Currently, the funding for the L&D fund is grossly inadequate to cover the estimated losses faced by developing countries, but it is hoped that more realistic levels of funding will be pledged in the future. Throughout 2023, a Transitional Committee carved out the broad parameters for how the fund might function, how it should be governed, and where it should be placed. Their recommendations were adopted at COP28, and a board will be set up in 2024 to further define how the fund will operate.

As the fund's operations will take shape over the coming years, actors will make decisions on whether resources should be disbursed, and, if so, how those resources should be spent. Both types of decisions call for a sound climate evidence base. The question of disbursement needs evidence to judge whether a given event, or activities in response to an event, are within scope of the fund, which ranges from “climate-related emergencies” to “climate-resilient reconstruction and recovery” (8). Indeed, the list of elements that the fund's board will consider when determining allocation of funding includes “considerations of the scale of impacts of particular climate events” (8). Consider an imaginary country that has been hit by torrential fluvial flooding, destroying critical infrastructure, costing many human lives, and requiring substantial recovery efforts. Reference to “particular climate events” indicates that the decision whether this country can receive financial support should consider whether anthropogenic global warming (AGW) contributed to the hazard and therefore to the damage. The second type of decision making determines how resources are spent. Should our imaginary country rebuild in the same area but install early warning systems and enforce building codes or should it shift infrastructure toward entirely new ground? Presuming that the policy goal is to reduce future L&D, evidence needs to inform actors on the biggest levers for risk reduction, and the potential need to shift from incremental to transformational changes.

Recent commentaries have argued both against and in favor of a strong role of event attribution science to support decisions on L&D (9, 10). While clearly the challenges are profound, we agree with Noy et al. (10) that we “should not wait for an imaginary future in which more improvements in extreme event attribution would make it perfectly suitable and scalable for the analysis of every type of extreme weather event, in every location.” Clearly, some kind of systematic and replicable framework is needed and it is needed now. Moreover, already today there is a strong climate evidence base, supported by many underlying attribution methods, for many types of extremes. However, what is being overlooked in this debate are the ethical implications that quantitative attribution studies can have. Addressing the ethical implications of decisions based on unevenly available data is essential (11). Lack of evidence for a role of AGW (in contrast to evidence for no role of AGW) can never solely serve as a ground to deny funding. These ethical implications are tied to the inherent uncertainty that is part of attribution of hazards and impacts, contingent on factors like the geographical region and the nature of the extreme event. A recent commentary appeared to dismiss uncertainty entirely (12), which is a wishful thinking approach. Uncertainty and its ethical implications cannot be ignored, and here we present a vision of how current methods can take these factors into account, and thereby play an essential role in L&D funding decisions.

## Informing decisions at the international level

**Fig. 1.**
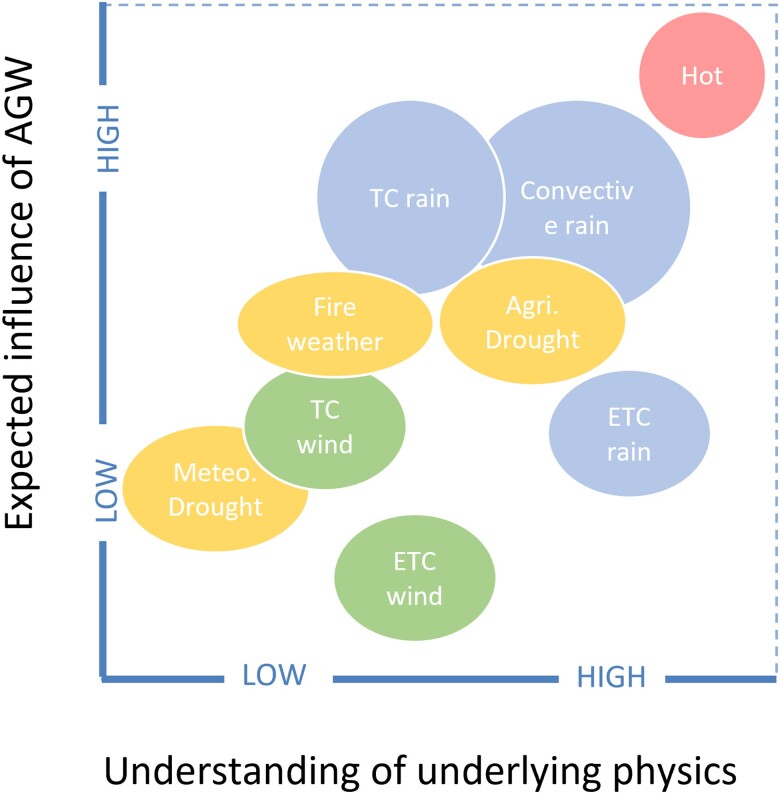
Qualitative assessment by authors (not based on an extensive literature analyses) of the generally expected influence of anthropogenic global warming on specific event types (vertical axis) against the understanding of the underlying physical processes (horizontal axis), see Box 1 for details. Extremes that are strongly influenced by thermodynamics cluster in the upper-right corner. Regionally, the positioning of event types can differ. Agri. Drought, agricultural or ecological drought (low soil-moisture levels/accounting for evaporation); ETC, extratropical cyclone; Meteo. Drought, meteorological drought (lack of precipitation); TC, tropical cyclone.

**Fig. 2.**
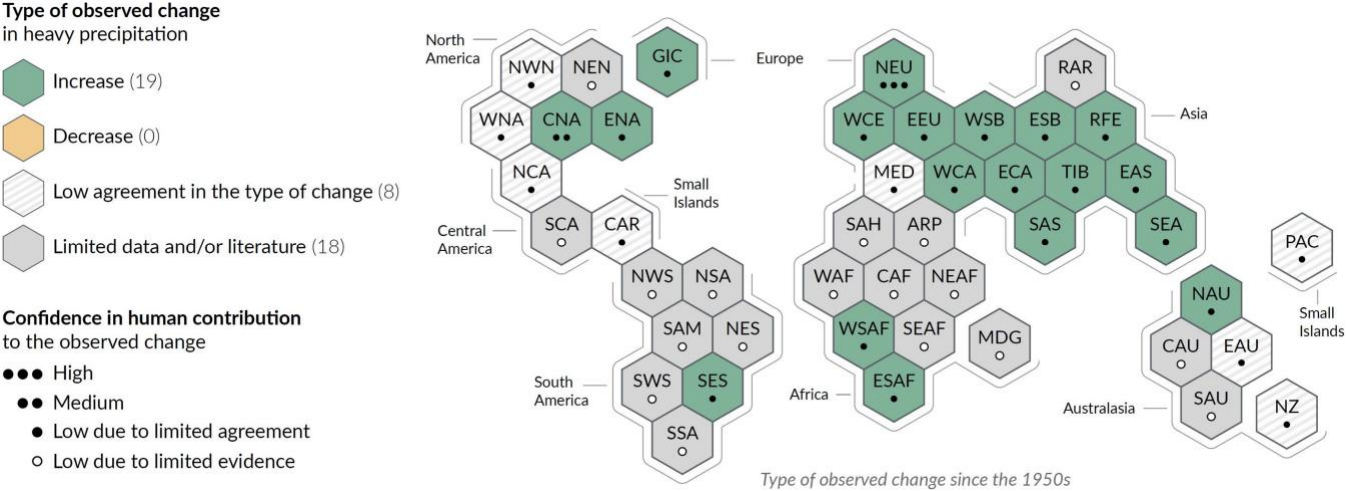
Synthesis of assessment of observed change in heavy precipitation since 1950 and the confidence in human contribution to the observed changes in the world's regions, adapted from the IPCC (1): Green regions have seen an increase, while gray regions, dominating in the tropics and southern hemisphere, show a lack of data or a lack of studies and thus a lack of quantitative evidence.

Decisions at the international level on whether or not to disburse funding need evidence to judge whether a given event, or activities in response to an event, are within scope of funding. We argue that decisions regarding in-scope or out-of-scope considerations should be grounded in the (qualitative) physical understanding of how AGW modifies specific hazards. The normative basis for this approach rests on considering greenhouse gas (GHG) emissions as a form of risk imposition, similar to a car above speeding limits hitting a pedestrian. Irrespective of whether the pedestrian could have avoided the risk of being hit, she is entitled to claims from the car driver. For emergency situations, a qualitative attribution statement based on a solid understanding of the physical processes and associated with an IPCC-style confidence assessment suffices. The previously mentioned IPCC statement—that AGW is already causing widespread impacts—rests on the robust physical understanding that global warming increases the intensity of those extremes that are closely linked to warming such as heat waves, heavy precipitation, increased fire weather, and evaporation-driven droughts (see Box 1, Fig. 1). Although there are regional variations (13), when aggregating over many such events, their expected rise in intensity is confirmed (1). Either a rapid qualitative attribution (within days) or ex ante information based on the scientific literature and general physical understanding could serve to identify which events fall within scope of funding under the L&D fund. From the ethical perspective mentioned previously, it is essential that this attribution is qualitative, based on literature and physics, rather than quantitative based on specific analyses of models or observations. To illustrate, the IPCC figure documenting changes in heavy precipitation (see Fig. 2) highlights large regions in the tropics and southern hemisphere (gray areas) that lack sufficient data to do quantitative detection and attribution analyses. Hence, quantitative observational analyses cannot provide evidence here for a role of AGW. Still, based on physics, supported by a large body of literature, we expect short-duration heavy precipitation to intensify everywhere. In fact, from basic thermodynamics (Clausius-Clapeyron equation) we expect a larger absolute increase in rainfall intensity per degree of regional warming in warmer tropical regions, as compared with the colder mid-latitudes. For our imagined country, this would mean that it could receive financial support from the L&D fund for emergency-related activities even if it is located within the gray-shaded regions in Figure 2. Ideally, this anticipatory, physics-based evaluation of various extreme events across diverse geographical areas is undertaken ex ante, drawing on entities like the IPCC, and integrated into a decision-making protocol for the L&D fund.

Box 1. **Attribution of different hazards.**

Understanding of damage-inducing weather systems and the influence of human-induced climate change on them varies across hazards, see Fig 1 (13). Increasing heat extremes on land and sea are confidently attributed to rising greenhouse gas levels (1). Locally, temperature extremes can be influenced by rainfall in the tropics (14), circulation changes (15), and human-induced aerosols. The Clausius-Clapeyron principle of increasing water holding capacity of warmer air (∼7% per degree) provides high confidence in the intensification of rainfall extremes due to GHG-induced warming (16). This applies to both tropical and extratropical storm systems (17, 18). In some locations, increasing vertical stability (19) and long-term drying counteract this effect (20). Several processes within convective rainfall extremes are thought to exhibit positive dynamical feedbacks with warming, and thus convective rainfall extremes are thought to intensify beyond Clausius-Clapeyron but the exact magnitude is uncertain (21). Drought metrics that include evaporation (e.g. agricultural drought) are attributable with medium confidence, due to the close link to warming, even if rainfall deficits are unchanged. Wind attribution studies show varying confidence levels, with the majority of studies analyzing tropical cyclones (e.g. 22). Attributing local wind intensity changes or major events to climate change requires an in-depth understanding of local factors, such as surface roughness, aerosols, and decadal variability (23). Circulation changes further complicate the picture, with some evidence indicating shifts in storm characteristics over the observed record (24). Both fire weather (hot and dry conditions) and length of the fire seasons have been increasing and are robustly projected to increase under warming in most regions (25). However, the actual occurrence of fire, the resulting impacts (burned area, biomass loss, carbon emissions), and their trends depends on both human and ecological factors influencing ignitions, fuel availability, and vulnerability to fire, which are not well simulated by current impact models (26).

## Informing decisions at the national level: multimethod hazard framework

The second type of decision making focuses on resource allocation at the national or community level. Presuming that the policy goal is to minimize future L&D, evidence is needed on the biggest levers for risk reduction, which requires quantitative analyses. This requires dealing with a range of uncertainties in a consistent way. First, extreme weather and extreme impacts are not always directly linked (27). This representational uncertainty depends on the fidelity of the climate models used to represent the events in question and the extent to which the attribution analysis is tailored to the impact-relevant aspects of the event. Here, quantitative compound metrics that are directly relevant to impacts are useful, including the Fire Weather Index, wet-bulb temperature, and agricultural drought. A second challenge is that attribution of the proximate causal factors behind extremes, such as anomalous large-scale atmospheric circulation regimes, may be unclear because of insufficient theoretical understanding and systematic biases in climate models (28). This epistemic uncertainty must be managed somehow, also because long-term trends in sea surface temperature (SST) patterns, sometimes outside the bounds of climate models predictions (e.g. 29), influence large-scale atmospheric circulation, teleconnections, and extremes (e.g. 15). These various uncertainties need to be acknowledged, but at the same time, one should not lose sight of those aspects of climate risks for which we have a robust understanding of the physical processes. To do so, we adopt the following Bayesian framing (e.g. 30):


(1) $$\frac{P_{f}(E,N)}{P_{c}(E,N)}=\frac{P_{f}(E|N)}{P_{c}(E|N)}\times \frac{P_{f}(N)}{P_{c}(N)}$$

The left-hand side term captures the probability ratio of extreme event *E* (e.g. a heat wave) together with a conditioning factor *N* conducive to that event (e.g. a blocking anticyclone), between the factual (subscript f) climate and a counterfactual (subscript c) climate without anthropogenic climate change. The uncertainty in the two terms on the right-hand side can be treated separately, reflecting their different levels of scientific confidence. By choosing the level of conditioning *N* appropriately, and nesting different causal factors (e.g. blocking anticyclones, SST anomalies, etc.), one can build causal accounts of the observed event that can meaningfully distinguish between the different kinds of uncertainty in a traceable way (31, 32).

**Fig. 3.**
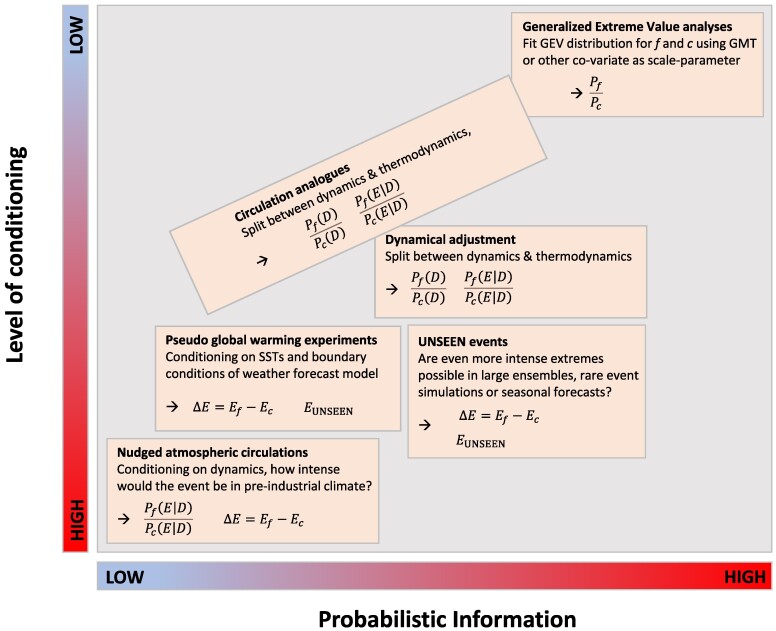
Different attribution methods, part of the multimethod hazard framework, schematically placed in terms of their level of conditioning (vertical axis) and amount of probabilistic information (horizontal axis).

The Bayesian equation forms the basis for our proposed multimethod hazard framework (Fig. 3), which consists of a stepwise approach with each step addressing subquestions of the quantitative attribution challenge by applying different levels of conditioning (N). The framework includes (i) highly conditioned storyline methods (e.g. nudged atmospheric circulations or pseudo global warming experiments) that enable conditional attribution statements, (ii) probabilistic methods with little conditioning that aim at estimating return times in present and preindustrial climate, and (iii) a range of methods that can bridge between (i) and (iii), including circulation analogues, dynamical adjustment techniques, and more. These methods enable to address a number of questions relevant for attribution:

1. Did AGW intensify this extreme event?

Storyline methods can quantify the intensity of an extreme under different background climates, with typically sharp uncertainty intervals due to high levels of conditioning, and provide estimates of the first term on the right hand side of Eq. 1. Nudged atmospheric circulation experiments (e.g. 33) can recreate an actual extreme event in present-day and counterfactual climates (preindustrial and/or future warm climates) and are particularly useful for extremes controlled by large-scale circulation (e.g. heat waves, drought). Such experiments condition on the large-scale circulation and thus primarily account for the role of thermodynamic effects in changing the event's characteristics. Likewise, pseudo global warming experiments use regional climate or weather models to reproduce individual extremes under different climatological background states by adapting the model's boundary conditions (e.g. 17). Such methods have been applied to quantify how AGW intensifies highly localized convective rainfall extremes (34) or tropical cyclones (35). Pseudo global warming experiments condition on the large-scale dynamics and thus also on remote teleconnection effects and associated SST patterns. However, regional and small-scale dynamical effects are resolved, including strengthening of convective storms due to enhanced latent heating, which can lead to super–Clausius-Clapeyron scaling of rainfall extremes (i.e. scaling beyond pure thermodynamic expectations).

2. What is the role of thermodynamic and large-scale dynamic processes in this extreme event?

Methods like dynamical adjustment techniques and analogue circulation analyses can be used to estimate the relative contribution of thermodynamic and large-scale dynamic aspects behind an event (24, 36). Thus, they form a natural extension of the storyline methods, enabling important cross-validation between different methodologies. While for many extremes the prime influence of climate change is thought to be through thermodynamic processes, it is imperative to quantify the potential influence of changes in large-scale circulation. Doing so provides estimates on the second term on the right-hand side of Eq. 1. Furthermore, these techniques are adept at quantifying different sources of uncertainty and discerning the roles of (multidecadal) natural variability versus anthropogenic forcing when applied to climate model output. Large model ensembles are required to establish statistical changes in circulation patterns, and numerous such datasets are becoming accessible (e.g. large ensemble seasonal hindcast experiments and large ensemble single forcing experiments, LESFMIP [Large Ensemble Single Forcing Model Intercomparison Project]). More information about the altered large-scale dynamics of the extreme is achieved through global coupled model forecasts with modified initial conditions, with altered GHG levels, that allow the background climate change state to influence the free progression of the extreme event (e.g. 19, 37, 38).

3. How much more intense could the event have been in current and future climates?

Using analogue circulation methods, one can also search large ensemble datasets to search for dynamically similar but even more-intense extreme events, or so-called UNSEEN extremes (39). Recently, rare event simulations and/or boosting ensemble experiments have become popular to create large samples of extremes, with even higher amplitude, in a computationally efficient way (40). This would assess high-end risks in both present and future climate, which is critical information for building resilience and adaptation. Some methods (i.e. rare event algorithms) are also able to provide estimates of return times of such worst-case extremes. Also, large ensemble seasonal forecasts can be used for attribution of observed and UNSEEN events.

4. How have return times changed for the extreme event?

Generalized extreme value (GEV) theory provides the basis to estimate return times in the tail of the distribution and can be applied to observations and climate model simulations (e.g. 41). Given sufficient data, GEV analyses can estimate the influence of global mean temperature on the return times of exceeding a specific extreme threshold. The level of conditioning is low but typically nonzero (e.g. often, the attribution statement is conditional on the SST patterns and counterfactuals are created by subtracting a GHG-forced SST anomaly from the actually observed SST patterns). GEV analyses provide estimates on the left-hand-side term of Eq. 1. However, when climate change changes the processes leading to extremes (for example, limiting the moisture supply not sampled in prior observations) or causes nonlinear trends, the result may be flawed as the underlying assumptions are violated.

These different attribution methods (Fig. 3) thus provide answers to different attribution questions that can be united, and cross-validated, using Eq. 1. This way, multiple lines of evidence can be integrated in a consistent logical framework, with clear estimates on uncertainty and confidence. It can inform decisions at the national level on how funding is best spent to reduce risk of future L&D. As an illustration of the multimethod attribution framework, Sippel et al (42). showed how multiple attribution methods, including dynamical adjustment, circulation analogues, rare event simulations, and GEV analyses, can be used in a complementary way. Analyzing cold extremes over Europe, they estimated the thermodynamic and dynamic contribution of warming, as well as the likelihood of extremely cold winters in the present and recent history, which together paint a consistent picture of the reducing cold risks over Europe due to AGW.

## From hazard to impact: incorporating local sources of information

Attribution of L&D, rather than of meteorological hazards, requires dealing with a range of challenges to do with the local scale of impacts introducing more sources of uncertainty. A first step toward impact attribution is to adopt quantitative metrics that are more directly relevant to impacts, such as agricultural drought instead of meteorological drought. From an impact perspective, it often does not matter whether water deficits are caused by lack of rainfall or increased evaporation. However, the latter has a much more direct link to anthropogenic global warming due to the increased water demand of warmer air. Similar arguments can be made for other impacts. Climate model representation of extremes is limited by their coarse resolution, which is often inadequate for resolving and locating relatively small spatial scale events. This is especially problematic when local topography, land cover, landscape heterogeneity, and other features play a key role. Statistical downscaling of extremes is limited by small observed sample sizes and the inherent stationarity assumptions. Global or regional kilometer-scale modeling can better resolve some events, most notably extreme precipitation (43). Uncertainty from the climate model and the downscaling propagates to impact models (44). Additionally, for proper quantification and attribution of the impacts, those models require realistic representation of local features, processes, and drivers that influence exposure (e.g. built environment, population density, land use) and vulnerability (e.g. social factors like poverty, education, employment, or ecological factors). Also, effective early warnings can significantly reduce the event's impact (45, 46). Realistic impact models thus strongly rely on a proper representation of such local conditions and human decisions, limiting their general applicability. More generally applicable impact models (e.g. global models in the Inter-Sectoral Impact Model Intercomparison Project) are often not realistic when applied at local scales due to their coarse representations and epistemic uncertainty. Noneconomic L&D (e.g. trauma, displacement, or loss of territory, biodiversity, or cultural heritage) are also dependent on local context and difficult to quantify or monetize (47). Drivers of exposure and vulnerability are complex and deeply rooted in historical, cultural, and institutional structures and paradigms. Sometimes, these have been historically conditioned (e.g. by colonialism), and in others, local governance structures play a more dominant role (see Boxes 2 and 3). Clearly, significant local knowledge, expertise, and context is required to defensibly represent local impact pathways and attribute the role of different factors in the event impacts.

Box 2. **Pakistan flooding 2022: climate change, bad governance, or colonialism?**

In 2022, Pakistan experienced flooding that submerged one-third of the country, affecting 33 million people, causing 1,700 casualties and a financial setback of $30 billion (48). The cascading economic effects materialized in 2023 when the nation grappled with its highest inflation in 47 years, which culminated in a food crisis (49).

The interplay of La Niña, a negative Indian Ocean Dipole and an anomalous subtropical jet stream, combined with a warmer atmosphere due to AGW, led to unprecedented rainfall. GEV-fitting analyses suggest that the rainfall is up to 50% more intense at present level of warming as compared with preindustrial (3). This estimate is highly uncertain due to large rainfall variability and limited observations, and observed changes could be attributed to various factors, including, but not limited to, climate change. Earlier in 2022, Pakistan and India witnessed a record-breaking compound hot-dry extreme in spring and early summer causing loss of lives, crops, livestock, and infrastructure (48). This early heat wave intensified a heat low, which deviated multiple monsoon depressions toward the northern provinces of Pakistan, resulting in 500% more precipitation than normal here (3). This heat wave, as all present-day heatwaves, is strongly attributable to climate change (50).

Pakistan, a lower middle-income country, is among the countries most at risk and least ready to tackle climate change (3). With several factors exacerbating the country's vulnerability such as geographical location and bad governance, its colonial history resulting in the present challenges make Pakistan a figurehead of climate injustice (51). Flood damages are not only caused by extreme weather, but also by infrastructure issues like dam backwater effects and failed irrigation levees (52). Engineering-driven interventions and irrigation systems, established during British rule, neglect local environment and equitable water distribution, hence contributing to the vulnerability of the communities. However, also the local government could be blamed for not taking enough precautionary measures after the devastating flood of 2010, which was a very similar event in terms of meteorological conditions, rainfall amounts, and flooding.

**Fig. 4.**
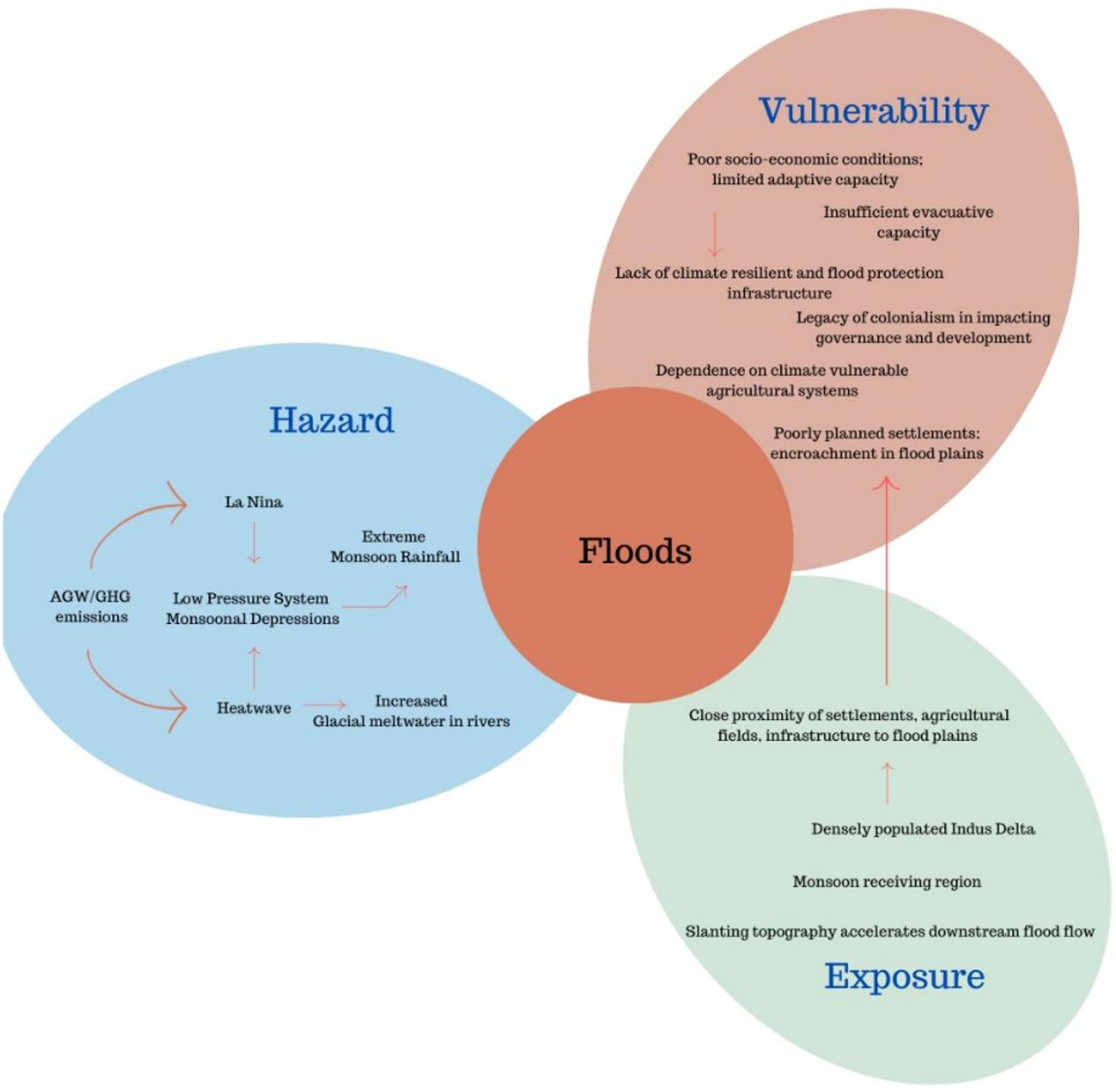
Schematic overview of the interplay between drivers of hazard, exposure, and vulnerability in driving the Pakistan flood disaster in 2022 (see Box 2 for details).

Thus, climate change, bad governance, and colonialism all contributed to the massive impacts experienced in the Pakistan flood of 2022, illustrating the complexity of disasters (see Fig. 4). Irrespective of the (perceived) responsibilities, storylines, enriched with local information, can help to design specific adaptation strategies to build resilience.

Box 3. **Multiyear drought in South America.**

**Fig. 5.**
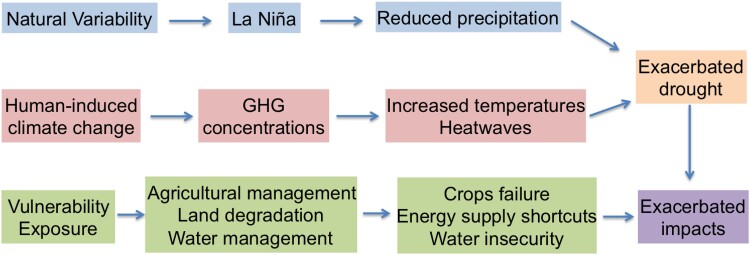
The interplay between natural variability, human-induced climate change, and local vulnerability and exposure behind the L&D that occurred due to the multiyear drought in South America during 2020 to 2023 (see Box 3 for details).

In recent years, Central South America experienced moderate to severe meteorological drought conditions, affecting extensive regions of Argentina, Paraguay, Uruguay, and Bolivia (5). A GEV-fitting analysis of the 2022 drought conditions, induced by 3 consecutive La Niña events in 2020 to 2023, shows that the observed rainfall deficit is within the range of natural variability. However, the impacts of the drought became much more severe due to simultaneous heat waves, which were strongly attributable to climate change (53). The impacts of this heat wave-exacerbated meteorological drought included the collapse of crop yields (worst crop health in 40 years), reduction of water access by city dwellers, and negative effects on fishing and navigation activities in local communities. Such impacts are worsened by nonclimatic drivers such as the land degradation experienced over 40% of farmland in Argentina, as well as water inaccessibility (54). The interplay between these different factors is shown in Fig. 5.

Attribution of this drought, like many events in developing countries, is challenging due to scarce meteorological in situ observations that provide long enough records to identify a robust signal. Moreover, general circulation models still exhibit important biases over South America, posing a great challenge for the attribution of extreme events and related L&D. Storyline approaches bypass both of those restrictions and thus constitute a good opportunity to deal with the interplay between the natural and human-induced drivers behind regional climate change.

How to deal with these cascading uncertainties? First of all, many societal risk-reduction measures are no regret and do not require detailed quantitative analysis. Such measures include enforcing proper building codes, and also poverty eradication, promoting gender equality, etc. Still, sometimes more fine-grained quantitative analysis of different drivers of risks is needed to estimate the potential and limits of different risk-reduction decisions. To do so, the multimethod hazard framework can be extended by local sources of knowledge, both quantitative and qualitative (e.g. local records or observations, Indigenous knowledge, anecdotal evidence and narratives) that can provide additional lines of evidence. Importantly, the Bayesian framework underpinning the multimethod approach allows for the incorporation of multiple lines of evidence. For example, participatory and community-based approaches provide opportunities for local stakeholders to identify features (e.g. land use planning, lack of infrastructure and maintenance) that contribute to cascading impacts, as well as assets and livelihoods at risk (55). Also, Indigenous observations of environmental parameters (e.g. changes in precipitation) have been integrated into adaptation efforts, as documented mostly in African and North American literature (56). Moreover, storylines (e.g. nudged atmospheric circulations or pseudo global warming experiments) enable flexible event definition in co-design with stakeholders and can integrate information on the hazard with local information on vulnerability and exposure to drive impact models to estimate L&D. This way, different drivers of vulnerability and exposure contributing to L&D can be assessed. The hazard framework can thus be a starting point to study societal drivers of change (recognizing historical contexts and culture which influence exposure and vulnerability factors) and assess the potential to reduce or avoid L&D by appropriate locally owned and governed adaptation actions. Here, also, UNSEEN extremes will add valuable information highlighting how intense an event could have been and thus what level of adaptation is advisable (39). A better understanding of the different climatic and societal drivers of impacts will help to prioritize risk reduction measures, providing important attribution information for recovery activities and adaptation and resilience building under the L&D fund.

## Community effort

Our vision on how attribution science can support decisions on L&D thus integrates qualitative physics, a range of quantitative methods to deal with hazard attribution, and detailed storyline approaches incorporating local information on vulnerability and exposure. A core component is to deal with uncertainty in a traceable way such that it becomes clear what information is robust and what information is not. It will be vital that studies follow peer-reviewed methods that are widely supported in the scientific community, and it will be important to convince a wide range of parties that the underlying tools are indeed fit for purpose. There is thus a clear role for umbrella organizations like the IPCC or World Climate Research Programme. These organizations could do a regularly updated, physics-based assessment to identify which type of extremes in which regions are within scope of the L&D fund to inform decision making at the international level. This could look like the map in Fig. 2, with underlying information for each region and event type. Such a framework would need to be nimble and rapidly updatable as new information arises, both within and outside climate research. World Climate Research Programme input can draw on the global research community, uniting experts from vulnerable developing countries with those in developed countries, and can be more nimble and forward looking. The multimethod hazard framework can integrate analyses performed at different institutes worldwide, and storylines of L&D should foster tighter collaboration with experts in vulnerable developing countries. Building local capacities for implementing this type of framework, both in terms of providing access and/or training researchers (e.g. impact modelers, disaster risk reduction) and in terms of training decision makers to appreciate a multiple-lines-of-evidence approach and grappling with deep uncertainties, may present a paradigm shift in many cases.

Performed ex ante, a physics-based assessment on eligibility led by a task force would immediately clarify whether a disaster is in scope or not for funding. Multimethod hazard attribution and vulnerability-enriched storylines on L&D would typically require a couple of months to several years to complete, depending on the level of operationalization and the complexity of the event and context. Technically, it would be fairly straightforward to operationalize the multimethod hazard framework. In fact, many methods are already used in semi-operational mode, including nudged atmospheric simulations, pseudo global warming experiments, analogue methods, and GEV fitting. With careful design, an operational platform would have numerous advantages including increased efficiency, transparency, reproducibility, and the facilitation of joint collaborative efforts between experts from developing and developed countries. Currently, scientists typically develop scripts and download data on local machines, hampering efficiency and reproducibility. Streamlining this workflow by migrating to central big data platforms not only fosters sharing and collaboration, but also significantly accelerates analyses across diverse datasets and methods. If event attribution science is to play a role in decisions on L&D funding, then transparency and reproducibility are key, and this can be safeguarded effectively on a central platform with open-source software. This would be of paramount importance for bolstering trust in attribution statements. Such a platform would yield an abundance of information, making it quite powerful. However, with any powerful tool, the key concern pertains to the manner in which this platform would be used and how the results are interpreted. As stated previously, large uncertainty (i.e. lack of evidence) should never solely serve as grounds to deny funding: uncertainty is inherently contingent upon the geographical region and the nature of the extreme event and can originate from issues of data availability, the proficiency of employed weather and climate models, and the scientific understanding of the dominant processes—factors that would disadvantage many regions that are particularly vulnerable to AGW.

Over time, such a platform could build an ever stronger evidence base, which could feed into the physics-based assessment, as well as identify important knowledge and data gaps. As new methodologies, like artificial intelligence, and new datasets, such as reanalysis or novel satellite products, become available, they can be relatively seamlessly integrated into existing workflows. Even short datasets can provide useful attribution information when interpreted in a conditional manner. The latest generation of high-resolution climate models (57, 58) shows promise in more accurately resolving extreme weather events. These improvements are particularly likely for small-scale rainfall extremes, but they may also enhance the representation of large-scale heat waves or droughts by better capturing remote teleconnections. Impact datasets pose greater challenges, as compared with climatic datasets, due to their diverse nature and the local scale of impacts. Nevertheless, numerous global or regional impact datasets exist, including EM-DAT, government-reported harvests, insured and noninsured losses, nongovernmental organization–reported casualties, and a range of satellite-derived products to estimate vegetation state, flooding, drought extent, wildfires, etc. The use of conditioning can again help extract robust information. In regions with a lack of proper observations, a purely model-based attribution using factual and counterfactual ensemble simulations would be informative, excluding an observational detection step altogether. The disadvantage here would be an even more strong emphasis on existing model biases, which can be large for several types of extremes.

## Conclusions

We have presented a vision on how the climate science community could support decision making on L&D funding, providing it an important climate science knowledge base. While attributing extreme events and their impacts is hampered by a range of challenges, many powerful methods exist, and there is a lot that is known about the role of climate change on extremes and disasters. Qualitative attribution statements, ideally made ex ante of events occurring, should inform the question of whether an event is within scope of funding. Further, our proposed multimethod hazard framework facilitates the integration of multiple lines of evidence on the quantitative influence of climate change on a particular extreme, to inform how resources can best be spent. This hazard framework can be extended toward impact attribution studies using storylines that integrate information on the hazard with local information on vulnerability and exposure together with stakeholders. Such studies would take longer but would provide clear policy recommendations on how to invest the longer-term L&D funding aimed at building resilience and reducing future risks. This could provide an important shift from incremental to transformative adaptation. With climate extremes hitting nations worldwide—and impacting vulnerable developing nations most severely—it is of utmost importance that resources under the L&D fund are disbursed swiftly and spent wisely.

## Data Availability

There are no data underlying this work.
